# Novel anatomical findings with implications on the etiology of the piriformis syndrome

**DOI:** 10.1007/s00276-022-03023-5

**Published:** 2022-09-29

**Authors:** Alexey Larionov, Peter Yotovski, Luis Filgueira

**Affiliations:** Faculty of Science and Medicine, Department of Oncology, Microbiology and Immunology (OMI), Anatomy, University of Fribourg, Route Albert- Gockel 1, CH-1700 Fribourg, Switzerland

**Keywords:** Piriformis, Deep gluteal space problems and piriformis syndrome, Sacral plexus, Pelvic pain syndrome, Pudendal neuralgia

## Abstract

**Purpose:**

The cause of the piriformis-related pelvic and extra-pelvic pain syndromes is still not well understood. Usually, the piriformis syndrome is seen as extra-pelvic sciatica caused by the entrapment of the sciatic nerve by the piriformis in its crossing through the greater sciatic foramen. However, the piriformis muscle may compress additional nerve structures in other regions and cause idiotypic pelvic pain, pelvic visceral pain, pudendal neuralgia, and pelvic organ dysfunction. There is still a lack of detailed description of the muscle origin, topography, and its possible relationships with the anterior branches of the sacral spinal nerves and with the sacral plexus. In this research, we aimed to characterize the topographic relationship of the piriformis with its surrounding anatomical structures, especially the anterior branches of the sacral spinal nerves and the sacral plexus in the pelvic cavity, as well as to estimate the possible role of anatomical piriformis variants in pelvic pain and extra-pelvic sciatica.

**Methods:**

Human cadaveric material was used accordingly to the Swiss Academy of Medical Science Guidelines adapted in 2021 and the Federal Act on Research involving Human Beings (Human Research ACT, HRA, status as 26, May 2021). All body donors gave written consent for using their bodies for teaching and research. 14 males and 26 females were included in this study. The age range varied from 64 to 97 years (mean 84 ± 10.7 years, median 88).

**Results:**

three variants of the sacral origin of the piriformis were found when referring to the relationship between the muscle and the anterior sacral foramen. Firstly, the medial muscle origin pattern and its complete covering of the anterior sacral foramen by the piriformis muscle is the most frequent anatomical variation (43% in males, 70% in females), probably with the most relevant clinical impact. This pattern may result in the compression of the anterior branches of the sacral spinal nerves when crossing the muscle.

**Conclusions:**

These new anatomical findings may provide a better understanding of the complex piriformis and pelvic pain syndromes due to compression of the sacral spinal nerves with their somatic or autonomous (parasympathetic) qualities when crossing the piriformis.

## Introduction

The piriformis is a flat isosceles triangle-shaped muscle of the gluteo-pelvic region contributing to hip extension, lateral hip rotation, hip abduction, and stabilizing of the hip joint [9, 31]. The origin of the muscle is variable. It may originate from three anatomical areas, including the pelvic/anterior surface of the sacrum, at the level of anterior sacral foramina 2,3 and 4, the gluteal surfaces of the ilium (close to the posterior inferior iliac spine), the sacrotuberous ligament, and the capsule of the sacroiliac joint. Its short narrow tendon attaches to the greater trochanter’s apex [2, 33]. The piriformis runs through the greater sciatic foramen but does not entirely fill it up, subdividing it into the upper and the lower slit, the suprapiriform, and the infrapiriform foramen. The suprapiriform foramen contains the superior gluteal vessels and the superior gluteal nerve, whereas the infrapiriform foramen contains the inferior gluteal vessels, the inferior gluteal nerve, the sciatic nerve, and the pudendal nerve [25, 33].

Innervation and blood supply of the piriformis are variable. The piriformis is innervated by the ventral rami of sacral plexus L5, S1, S2 [2, 11]. Different innervation patterns have been described, including innervation from the caudal-most root of the superior gluteal nerve, the inferior gluteal nerve’s caudal roots, and the sciatic nerve portion of the common peroneal nerve[1]. In addition, the blood supply may derive from the branches of the inferior and superior gluteal artery [17], internal pudendal, and lateral sacral artery [2, 7].

The piriformis has a variable morphology. The muscle can be identified either as an undivided (entire) muscle in one block or with several bellies or completely separated sub-units as accessory independent muscles. Accessory bellies may be present mainly when originating from the sacrotuberous ligament or the sacrotuberous fascia overlying the gluteus medius [26]. It is often tightly associated with neighboring muscles, including the gluteus minimus, the gluteus medius, the gemellus superior, and the obturator internus, and it may be difficult to clearly separate the borders through anatomical dissection or medical imaging techniques [6]. The origin of the piriformis on the sacrum is variable and includes: (1) the classical sacral type (muscle originates between the 2nd and 4th anterior sacral foramina); (2) the supplementary sacral type (slip to the first sacral segment); (3) the reduced sacral type (attachment to two sacral segments); (4) the coccyx attachment; (5) the atopic type (rare variation according to the literature), where the muscle starts as one long slip medial to the ventral sacral foramina and is pierced by grey sympathetic rami and branches of the lateral sacral artery [19, 33].

The topography of the piriformis is complex and has been well-studied in the gluteal region but to a lesser degree in the pelvic cavity within the minor pelvis before crossing the greater sciatic foramen. The gluteal surface of the piriformis is covered by the gluteus maximus and may be partially covered by the gluteus medius and/or the gluteus minimus. The superior gemellus may be tightly connected to the piriformis, and in some cases, it impedes a clear separation between the two muscles, making the identification of the lower border of the piriformis difficult [20]. Imbalanced topography of the piriformis muscle and the sciatic nerve is a predisposition factor for the piriformis syndrome initiation. Clinically, this syndrome is a part of deep gluteal space problems, including the neuro-muscular disorders of the obturator internus, the gemellus, the quadratus femoris, and the gluteal muscles [22]. Piriformis syndrome is usually associated with sciatic nerve entrapment by the muscle. The topography between the piriformis and the sciatic nerve in the gluteal area has been well described and includes around six different anatomical variations: (1) the unsplit sciatic nerve below the entire muscle; (2) the divisions of the sciatic nerve with one portion within and one portion below undivided piriformis; (3) the divisions of the sciatic nerve with one portion above and one portion below the undivided piriformis; (4) the undivided sciatic nerve between the heads of the piriformis; (5) the nerve divisions between and above heads of the piriformis; (6) the unsplit sciatic nerve above the undivided piriformis [23, 35, 37]. In the pelvic cavity, the piriformis muscle is adjacent to the sacral plexus, pelvic organs, and vessels. However, the detailed relations between the piriformis, the sacrum, the sacroiliac joint, and the ventral branches of the sacral spinal nerves are still poorly understood. Variations of the neuro-muscular spacing in the pelvic cavity (not in the gluteal area) may trigger inflammation of the piriformis, affecting the pelvic organs, as well as somatic and autonomous nerves.

This work proposes a new anatomy-based explanation of the development of pelvic pain syndromes. The interrelation between the piriformis and adjacent structures forming the sacral plexus are highly variable and was thus accordingly studied in detail. Our hypothesis postulates that deep gluteal space problems, particularly the piriformis syndrome, primary and idiopathic pelvic pain syndrome, pudendal neuralgia, dysfunctional pathologies of the pelvic organs, or degenerative diseases of pelvic muscles may result from the compression of the sacral nerves at its sacral origin by the piriformis or damaged sacroiliac joint in the pelvic cavity. The innate topographic relationship in the pelvis and the entrapment of nerves crossing the piriformis may be predisposition factors for corresponding pelvic conditions. Topography of the S1 spinal branches of the lumbosacral plexus and sacroiliac joint may be another risk factor for pelvic pain syndromes (data are not shown here) too. The new findings of the piriformis anatomy and sacroiliac joint topography must be considered in the context of diagnosing deep gluteal and particularly piriformis syndromes, pelvic pain in surgery, gynecology, and orthopedics. However, the vascular component in the initiation of gluteal and piriformis syndromes formation should not be excluded. Williams et al. postulate a vascular hypothesis for the development of piriformis syndrome development. They propose that a pain syndrome with irradiation into the posterior thigh may be due to varicosities of the inferior gluteal vein leading to irritation of the posterior femoral nerve (vascular compression) [38].

## Methods and materials

### Gross anatomy

The anatomy of the piriformis and its topographic relationships with the anterior branches of the sacral spinal nerves forming the sacral plexus were investigated on 14 paired (hemipelvises from the same person, 11 female, and 3 male) and 12 unpaired hemipelvises (hemipelvises from different persons, 4 female, and 8 male), in totally 40 unpaired hemipelvises with attached lumbar region and leg, used in the gross anatomy dissection courses for medical students at the University of Fribourg. Human cadaveric material was used accordingly to the Swiss Academy of Medical Science Guidelines adapted in 2021 and the Federal Act on Research involving Human Beings (Human Research ACT, HRA, status as 26, May 2021). All body donors gave written consent for using their bodies for teaching and research. 14 males and 26 females were included in this study. The age range varied from 64 to 97 years (mean 84 ± 10.7 years, median 88). The exclusion criteria were surgery and trauma of the gluteal or pelvic regions. The bodies were embalmed according to the Jores protocol [15]. Standard anatomical dissection methods were applied. First, the gluteal region was dissected. The skin, the fascia lata, and the superficial gluteal fascia were removed. The gluteus maximus was detached from its origin, and the piriformis was exposed together with adjacent vascular and nerve structures. Secondly, the morpho-topographic investigation of the piriformis's internal/anterior/pelvic surface and adjacent nerve structures in the minor pelvic cavity was done. For this purpose, the internal pelvic organs were removed without damaging the piriformis and the lumbar and sacral plexuses. We investigated the piriformis origin and its relationships with the lumbosacral plexus nerves on all hemipelvises, as well as in comparison with the paired specimens. The dissection was photo-documented step-by-step. Findings were accordingly documented following a previously elaborated protocol.

### Statistical analysis

Statistical analysis was done with GraphPad Prism 9.1.1 software. To facilitate data representation, we made all calculations for the total amount of hemipelvises, 40 specimens.

## Results

**Fig. 1:**
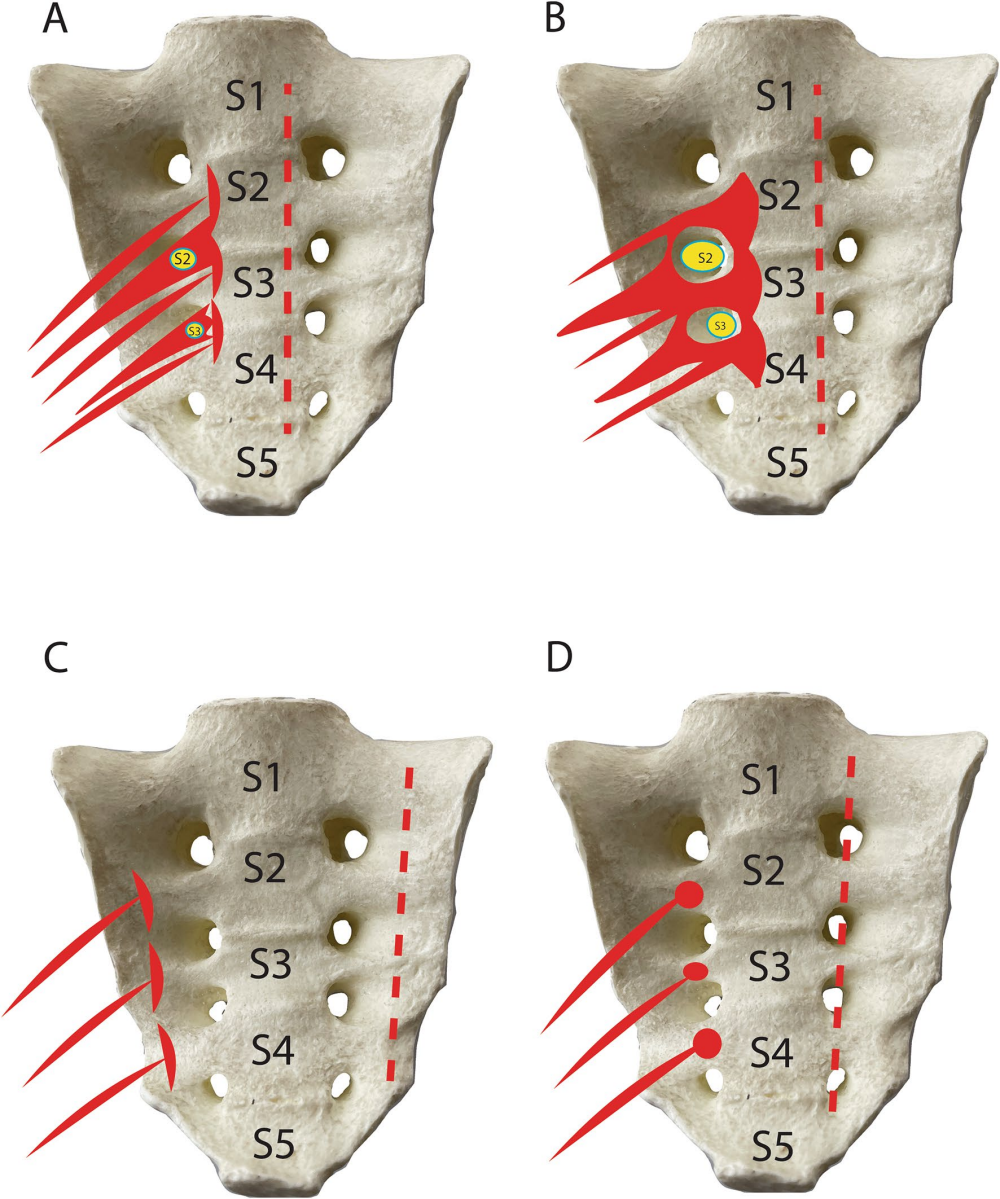
Schematic representation of the patterns for the piriformis origin. **A** Medial pattern with nerve entrapment; **B** medial pattern without nerve entrapment; **C** lateral pattern, and **D** interforaminal pattern (detailed description see in the text). The border of muscle origin is shown on the opposite side with the dashed line:** A** and **B** medially to sacral foramina, **C** laterally to sacral foramina, **D** in the middle of interforaminal space (interforaminal position)

40 hemipelvises (14 paired and 12 unpaired hemipelvises, among them, 14 males (3 pairs and 8 unpaired) and 26 females (11 paired and four unpaired) were investigated to identify morphological variants of the piriformis origin and its topographic relationships with the anterior branches of the sacral spinal nerves forming the lumbosacral plexuses. According to published information, the piriformis originated laterally around four anterior sacral foramina [6]. However, our dissection and observation exposed three patterns of muscle origin with respect to the anterior sacral foramina: i.e., a medial, an interforaminal, and a lateral origin (Fig. 1A–D).

**Fig. 2:**
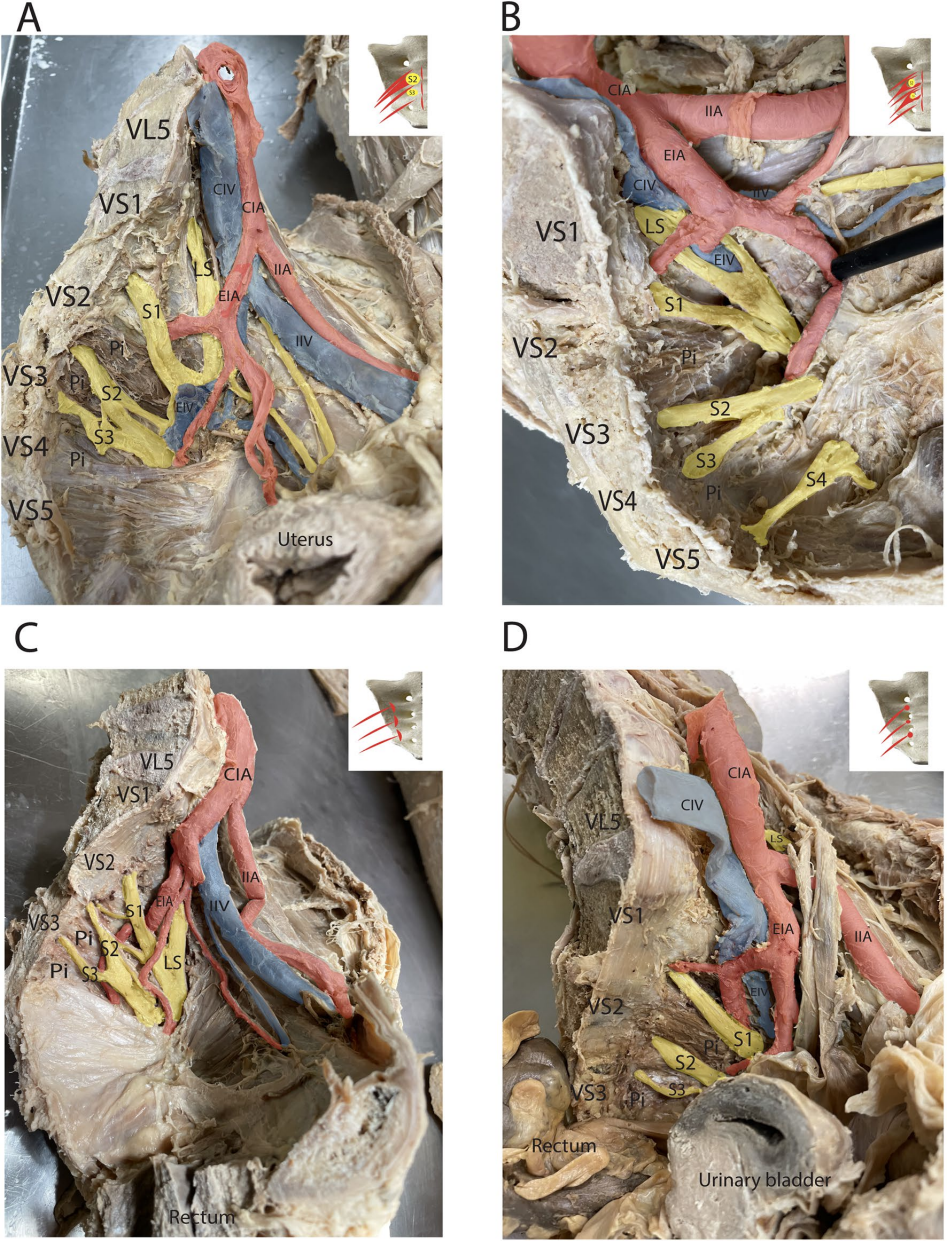
Examples of the main patterns for piriformis origin on corresponding anatomical specimens. **A** Medial pattern with nerve entrapment; **B** the medial pattern without nerve entrapment; **C** lateral pattern, and **D** interforaminal pattern (explanation see in the text).*CIA* common iliac artery, *CIV* common iliac vein, *EIA* external iliac artery, *IIA* internal iliac artery, *IIV* internal iliac vein, *S1* ventral branches of the sacral spinal nerves 1, *S2* ventral branches of the sacral spinal nerves 2, *S3* ventral branches of the sacral spinal nerves 3, *S4* ventral branches of the sacral spinal nerves 4, *S5* ventral branches of the sacral spinal nerves 5, *LS* lumbosacral plexus, *VL5* lumbar vertebrae 5; *VS1* sacral vertebrae 1, *VS2* sacral vertebrae 2, *VS3* sacral vertebrae 3, *VS4* sacral vertebrae 4, *VS5* sacral vertebrae 5

The medial pattern was allocated when the muscle’s origin began medial to the anterior sacral foramina. In this case, the muscle either covered the foraminal opening entirely with enwrapping the nerves, or it followed the medial peripheral border of the anterior sacral foramina allowing open passage of the nerves (Figs. 1A, 2B). The medial pattern was observed in as many as 75% (*n* = 30/40) of all examined hemipelvises. The lateral pattern represented the piriformis origin lateral to the anterior sacral foramina (Fig. 1C) with a frequency of 10% (*n* = 4/40). The interforaminal pattern revealed the muscle origin at the level and between the anterior sacral foramina (usually the second and third foramen) (Fig. 1D). The interforaminal origin of the piriformis was confirmed in 15% (*n* = 6/40) of cases.

The topography of the piriformis and its relationship with the anterior branches of the sacral spinal nerves forming the sacral plexus in the pelvis may be clinically relevant. Herein, we distinguished four types of topographic relationships, based on the piriformis origin and also considering the crossing of the ventral branches of the sacral spinal nerves before forming the sacral plexus: (1) the medial piriformis pattern with the ventral branches of the spinal nerves enclosed by the piriformis (medial pattern with nerve entrapment); (2) the medial piriformis pattern without the piriformis enclosing the nerves (medial pattern without nerve entrapment); (3) the lateral piriformis pattern with a free course of the nerves, and (4) the interforaminal piriformis pattern with the free course of the nerves (Table 1).

**Table 1:** Frequency of piriformis variations: muscle origin

Medial pattern (*origin medial to anterior sacral foramina***)** (total *n* = 40)		Lateral pattern (*origin lateral to anterior sacral foramina*) (total *n* = 40)	Interforaminal pattern (*origin between anterior sacral foramina)* (total *n* = 40)
Medial pattern with nerve entrapment	Medial pattern without nerve entrapment		
60% (*n* = 24)	15% (*n* = 6)	10% (*n* = 4)	15% (*n* = 6)

**Fig. 3:**
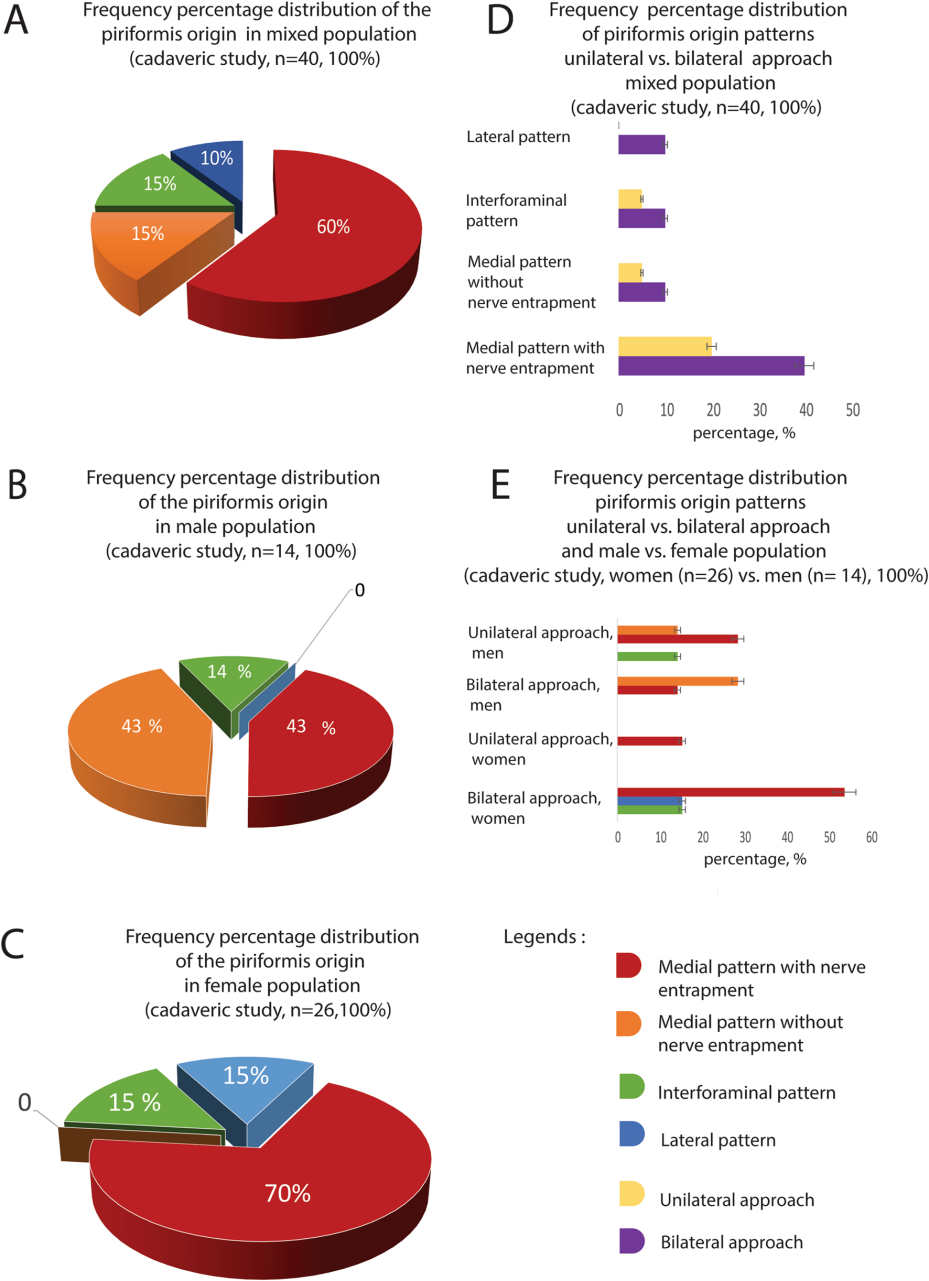
Frequency of the piriformis origin patterns. **A** Frequency of piriformis origin patterns in total (*n* = 40); **B** frequency of piriformis origin patterns in males (*n* = 16); **C** frequency of piriformis origin patterns in females (*n* = 24)

In 60% (*n* = 24/40) of cases, the S2 (55%, *n* = 24/40) and S3 (5%, *n* = 2/40) ventral branches of the sacral spinal nerve were entrapped by the piriformis (Figs. 2A, 3A).

Routine dissection displayed that the S2/S3 nerves might be entrapped completely or partially. Interestingly, the S1 ventral branch was never compressed by piriformis. The medial pattern without nerve entrapment (15%, *n* = 6/40, Figs. 2B, 3A) together with the lateral (10%, *n* = 4/40, Figs. 2C, 3A), and the interforaminal (15%, *n* = 6/40, Figs. 2D, 3A) patterns consisted of 40% (*n* = 16/40) of dissected cadaveric specimens without a tight muscle -nerve interaction. Of note, the piriformis had no topographic affiliation with the lumbosacral trunk.

Topographically it was interesting to compare the distribution of the various patterns of the piriformis origin in paired (i.e., from the same individual) vs. impaired hemipelvises. This approach was important for the estimation of left and right side symmetry. This comparison revealed that bilaterally the medial piriformis pattern with nerve entrapment was found in 40% (*n* = 16/40) of all cases, unilaterally only in 20% (*n* = 8/40).

Bilaterally, the medial pattern without muscle entrapment and interforaminal patterns were distinguished in 10% (*n* = 4/40). Unilaterally, in 5% (*n* = 2/40) unilaterally (Fig. 3 D).

In addition, we also investigated the innervation of the muscle itself and described four innervation variants of the piriformis from the lumbosacral plexus (Table 2).

**Table 2:** Innervation pattern of piriformis from branches of spinal nerves

Innervation of periformis (total *n* = 40)	S1	S2	S1 + S2	Lumbosacral trankc -L5
	40% (*n* = 16)	20% (*n* = 8)	20% (*n* = 8)	20% (*n* = 8)

40% (*n* = 16/40) of all hemipelvises received innervation from the first sacral plexus root (S1). The lumbosacral fibers L-4/L5 provided innervation to the piriformis in 20% (*n* = 8/40). The second sacral plexus root (S2) sent branches to the piriformis in 20% (*n* = 8/40). Finally, double innervation from S1 and S2 sacral plexus roots was found in 20% (n = 8/40).

Analysis of the gender distribution of the described neuro-muscular topographic patterns revealed that the medial pattern with nerve entrapment was more often observed in females 70% (*n* = 18/26) than in males 43% (*n* = 6/14). Medial pattern without nerve entrapment was not registered in females (*n* = 0) but was often observed in males 43% (*n* = 6/14). The lateral pattern was not detected in males but was found in 15% of the female population (*n* = 4/26) (Fig. 3B, C). The interforaminal pattern was observed slightly more often in women (15%, *n* = 4/26) than in males (14%, *n* = 2/14).

The bilateral approach and the gender analysis showed that the frequency of medial pattern with nerve entrapment on both pelvic sides was relatively high in the female population and reached 54% (*n* = 14/26) compared with 14% (*n* = 2/14) in the male population. (Fig. 3E).

## Discussion

For several decades, the piriformis has remained a muscle of clinical and scientific inquisitiveness. Scientific interest is based on topographic relationships of this muscle with the neuro-muscular and splanchnologic environment in the pelvic region. Clinicians are interested in the role of piriformis in piriformis syndrome development. Nosologically, the piriformis syndrome belongs to the group of deep gluteal space problems. In medical practice, the complexity of the piriformis syndrome requires a differential diagnosis that includes sacroiliac joint dysfunction [8], wallet neuritis, leg-length discrepancy [32], myofascial gluteal pain syndrome, trochanteric bursitis, the facet syndrome, etc. Unfortunately, the lack of anatomo-topographic data about piriformis frequently results in misunderstandings of pelvic problems related to this muscle.

The current research focused mainly on the piriformis anatomy and topography, with the muscle’s role in the development of piriformis-associated pathologies and pain syndromes of the pelvic region: deep gluteal space problems, piriformis syndrome-sciatic neuropathy, piriformis syndrome—sacroiliac joint disorder. Our patho-etiological concept is based on detailed anatomo-topographic findings during routine cadaveric dissection. It is the first anatomical investigation of the relationship between the piriformis origin and the adjacent anterior branches of the sacral spinal nerves, which extends R.Robinsons’ 1947 work on the piriformis syndrome [27].

**Fig. 4:**
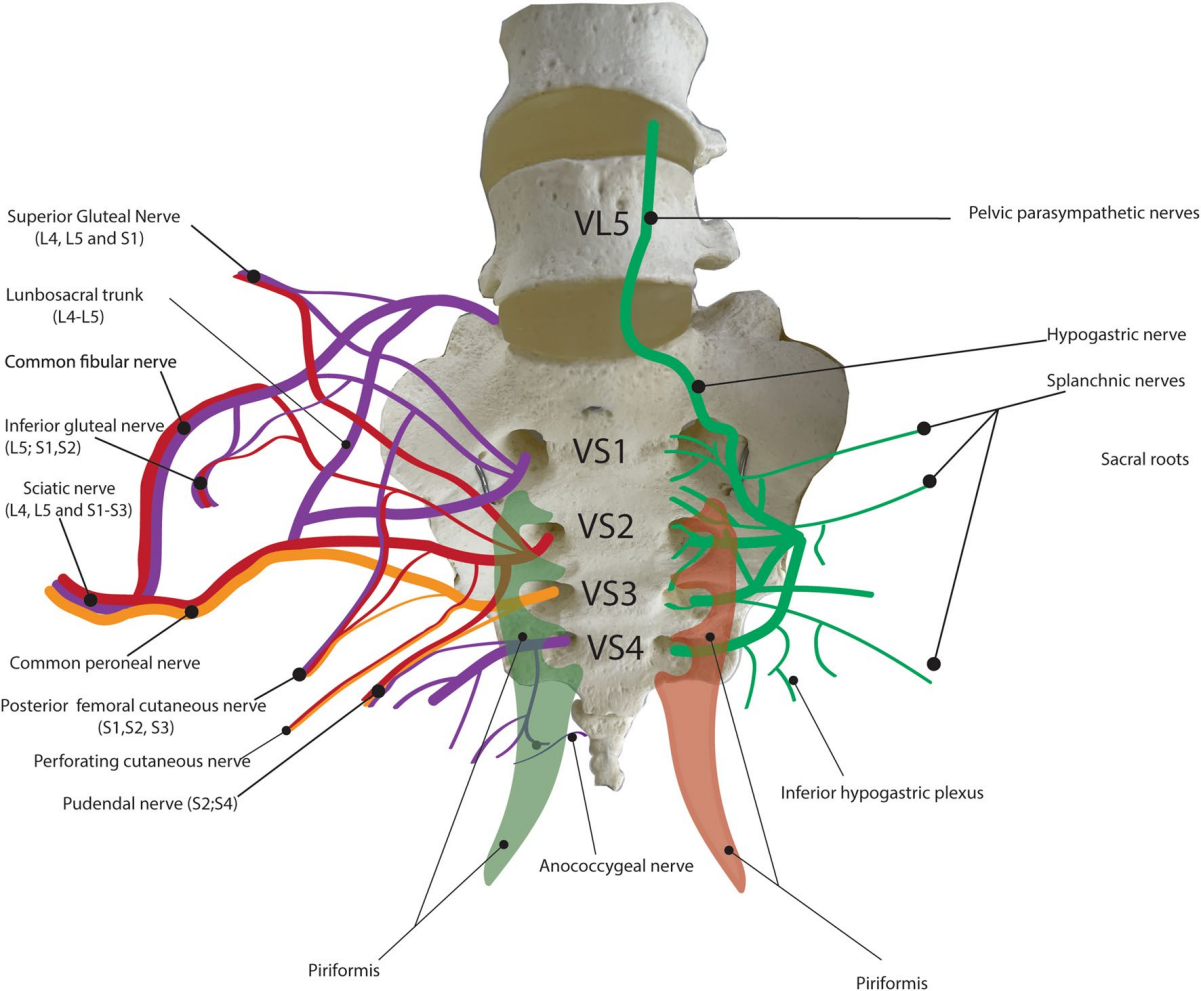
Schematic drawing of topographic relationship between the piriformis and the peripheral nervous network in the pelvis.VL5- vertebra lumbalis 5; VS(1–5)- vertenrae sacralis 1–5; S(1–5) sacral segments of sacral plexus

Our revision of the piriformis anatomy (muscle morphology, topography, and innervation) through dissection identified four neuro-muscular topographic patterns of the piriformis origin in the pelvis which have not been described before, such as (1) the medial pattern with entrapment of the anterior branch of the sacral spinal nerves); (2) the medial pattern without nerve entrapment; (3) the lateral piriformis pattern with the free course of the nerves, and (4) the interforaminal piriformis pattern with the free course of the nerves. The medial pattern of the piriformis origin was found in 75% of the studied cadaveric hemipelvises and could be regarded as an essential innate predisposition factor for pelvic and extra pelvic pain. Interestingly, 60% of all cases with medial muscle origin are characterized by entrapment of S2 ventral branches of the sacral spinal nerve. The nerve entrapment is thus characterized by: (1) the attachment of the piriformis to the second anterior sacral foramina's medial border and (2) a complete covering and filling up of the corresponding foramen. In such cases, muscle trauma, spasms, hypertrophy, and inflammation may enhance piriformis constriction and nerve compression. The S2 root of the sacral plexus, which is the most frequently affected segment, sends the fibers to somatic and autonomic nerves of the pelvis and the leg (Fig. 4), including the sciatic nerve, the tibial nerve, the common fibular nerve, the pudendal nerve, the inferior and superior gluteal nerves, the femoral nerve (only partly from the sacral plexus, most from the lumbar plexus), the posterior posterior femoral cutaneous nerves [2, 14]. S2 participates in the innervation of the obturator internus, the piriformis and the gemellus superior muscles [2, 20], and is potentially responsible for the initiation and progression of deep gluteal syndrome.

Additionally, compression, damage, or ischemia of the S2 segment may be responsible for developing piriformis syndrome, as well as pelvic and extra pelvic pain (idiopathic, primary), including pudendal neuralgia, pelvic organ dysfunction, and andro-gynecological and urological disorders (Fig. 4). According to our findings, the gender dependency of the piriformis distribution points out that the female gender may be at higher risk for pelvic pain and deep gluteal syndrome evolution. Interesting, mainly in the female population, piriformis origin and topographic relations with the nerves occurred bilaterally. The medial pattern with nerve entrapment was found in more than half of the studied female cadaveric paired hemipelvises, while the majority of the male cohort demonstrated unilateral dispositions of the described pattern (1/3 of the male cohort). These findings agree with Kapor et al. publication, which demonstrated that the female sex predisposes pelvic pain syndrome to evolution [16]. The main reason for this is the anatomic specificity of women’s pelvis with larger dimensions (wider “Q angle”) vs. men's pelvis, which increases the incidence of piriformis syndrome.

Studies about the origin of piriformis were previously done. However, the variations of piriformis origin and corresponding topographical relationships with the anterior branches of the sacral spinal nerves were not yet considered [19]. Prior anatomical studies have reported that the topographic relationship between the sciatic nerve and the piriformis was responsible for sciatic nerve compression and thus the piriformis syndrome [12]. Beaton and colleagues, i.g., were the first to describe the variable topography of the sciatic nerve and the piriformis muscle and suggested the potential role of the variations in the initiation of the piriformis syndrome [5]. However, this concept of piriformis syndrome is relatively narrow. First, the piriformis muscle is also located in the pelvic region (the muscle has its origin at the internal pelvic surface of the sacrum and close to the anterior sacral foramina) and not exclusively in the gluteal region (muscle attaches in this region to the apex of the greater trochanter). Second, in addition to its topography related to the sciatic nerve in the gluteal region, the piriformis also has a complex relationship with the pelvic organs, the pelvic nerves, and vessels. The piriformis syndrome is classified as a primary (muscle and nerve abnormalities, individual sciatic nerve path) or a secondary (resulting from muscle and nerve trauma) condition, with or without nerve entrapment (entrapment neuropathy). None of the piriformis syndrome forms are related to prolapsed intervertebral discs or herniated nucleus pulposus [29]. They are associated with muscle and joint pathologies, overloading the lower back and buttock [36], and proceed as a false (pseudo-) sciatica syndrome. The piriformis syndrome with compression of the sciatic nerve occurs quite rarely, just in 6–8% of all sciatica cases [24]. Numerous anatomical reviews suggested that the piriformis may be a trigger (in case of entrapment neuropathy) [3, 11] or a “victim” of several pelvic pathologies [10, 21, 39]. That is why the concept of piriformis syndrome may not exclusively be associated with sciatic nerve pain. Several publications speculated about the role of sacroiliac joint arthritis as a trigger of the piriformis syndrome [10, 28, 34, 39]. This point of view is worth the attention of anatomists and clinicians because it may explain unusual sciatica cases and piriformis syndrome without sciatic nerve entrapment. During routine dissections, we observed that the piriformis had no direct contact with a sacroiliac joint. The piriformis axis (a line from the cranial muscle origin or the sacral foramina to the muscle attachment on the greater trochanter) was located 0.5–1,5 cm caudally of the sacroiliac joint (data not shown). However, L5 and S1 nerves were located close to the sacroiliac joint (in some cases, the nerves were located above the sacroiliac joint; in other cases, they just crossed the sacroiliac joint at its lower third but always entered into contact with the sacroiliac joint anterior projection, data not shown). Therefore, we suppose that sacroiliac joint traumatism, post-traumatic conditions, and any inflammation processes may elicit neurological effects on the L5–S1 anterior spinal branch. It is also may be extrapolated on all nerves originating from the L5—L4–S1 nerve, e.g.for, the superior gluteal nerve. Interestingly, S1 ventral branches innervating the piriformis in 40% (*n* = 16/40) were located at the area of sacroiliac joint projection. This fact may explain the phenomena of piriformis pain, posterolateral gluteal pain, and any unusual pain in the buttock area without sciatic nerve compression. Inflammation or degenerative processes in the sacroiliac joint may lead to L5-S1 irritation, provoke piriformis contraction (if muscle receives innervation from S1 branches), and the development of false sciatica. Further, the irritated piriformis may compress the S2 anterior branch of the sacral spinal nerve (medial pattern nerve entrapment), leading to aggravation of symptoms and an uncontrol pain syndrome. In the opposite situation, the sacroiliac joint problems may lead to sciatic nerve inflammation (if the S1 fibers were damaged by the sacroiliac joint; the sciatic nerve is composed of the L4 to S3 fibers) with intact piriformis muscle [4, 18, 34].

Through clinical observations, Sermer and colleagues anticipated our findings and gave an anatomical explanation of the anterior branches of the sacral spinal nerve compression by the piriformis in the pelvis [30]. They describe eight clinical observations of intrapelvic entrapment of sacral nerve roots by abnormal bundles of the piriformis treated with the laparoscopic neuronavigation technique. However, we must emphasize that abnormal bundles of the piriformis muscle (described in Sermer’s article) should be put in the context of the piriformis origin patterns as described in detail in this work. Akita and colleagues described the piriformis innervation by branches of the superior gluteal nerve [1]. Our dissection has not revealed this innervation pattern. However, we suppose this variation is highly probable because the superior gluteal nerve receives branches from L4-L5-S1 portions of the lumbosacral plexus. Additionally, our findings are in concordance with the results of J. Iwanaga et al., who demonstrated that the piriformis, in most cases, receives innervation from the S1 sacral plexus root [13].

This research accentuates the topographic relationships between the piriformis and the anterior branch of the sacral S2 spinal nerve as a possible reason for initiating and developing pelvic pain and the deep gluteal piriformis syndromes in male and female populations. Additionally, the anatomy of the S1 ventral branch and the sacroiliac joint was revised (data are not shown here), and the fallacious topographical relationships between the S1 and sacroiliac joint were found (data are not shown here). The neighboring of the S1 sacral branches and sacroiliac joint may potentially predispose to damage of S1 and its nerves and participate in the development of false sciatica, deep gluteal, and piriformis syndromes [38].

Finally, as part of deep gluteal space problems, piriformis syndrome has a complex nosology. Future work may include detailed clinical re-examinations of the primary reasons for pelvic pain, pudendal neuralgia, and andro-gynecological and urological disorders, considering and integrating our anatomo-topographic findings to improve patient quality of life by possibly establishing new surgical and other related therapies.

## Conclusion

Our study revealed that the medial pattern of origin of the piriformis is a frequent anatomical variation with a possible predisposition for the development of deep gluteal, piriformis, and pelvic pain syndromes. Furthermore, the ventral branches of the sacral S2 spinal nerves leaving the S2 sacral foramen may be easily entrapped by the corresponding piriformis variation. Therefore, any piriformis condition around the anterior sacral foramina with swelling and inflammation should be regarded as a potential pathological trigger of the piriformis and pelvic pain syndromes related to S2 ventral branches.

## Data Availability

All data used in this work are available from the corresponding author on reasonable request.
